# TRH Analog, Taltirelin Protects Dopaminergic Neurons From Neurotoxicity of MPTP and Rotenone

**DOI:** 10.3389/fncel.2018.00485

**Published:** 2018-12-20

**Authors:** Cong Zheng, Guiqin Chen, Yang Tan, Weiqi Zeng, Qiwei Peng, Ji Wang, Chi Cheng, Xiaoman Yang, Shuke Nie, Yan Xu, Zhentao Zhang, Stella M. Papa, Keqiang Ye, Xuebing Cao

**Affiliations:** ^1^Department of Neurology, Union Hospital, Tongji Medical College, Huazhong University of Science and Technology, Wuhan, China; ^2^Department of Neurology, Renmin Hospital, Wuhan University, Wuhan, China; ^3^Yerkes National Primate Research Center, Emory University School of Medicine, Atlanta, GA, United States; ^4^Department of Neurology, Emory University School of Medicine, Atlanta, GA, United States; ^5^Department of Pathology and Laboratory Medicine, Emory University School of Medicine, Atlanta, GA, United States

**Keywords:** Parkinson’s disease, TRH, taltirelin, MPTP, rotenone, tau, α-synuclein, AEP

## Abstract

Dopaminergic neurons loss is one of the main pathological characters of Parkinson’s disease (PD), while no suitable neuroprotective agents have been in clinical use. Thyrotropin-releasing hormone (TRH) and its analogs protect neurons from ischemia and various cytotoxins, but whether the effect also applies in PD models remain unclear. Here, we showed that Taltirelin, a long-acting TRH analog, exhibited the neuroprotective effect in both cellular and animal models of PD. The *in vitro* study demonstrated that Taltirelin (5 μM) reduced the generation of reactive oxygen species (ROS) induced by MPP+ or rotenone, alleviated apoptosis and rescued the viability of SH-SY5Y cells and rat primary midbrain neurons. Interestingly, SH-SY5Y cells treated with Taltirelin also displayed lower level of p-tau (S396) and asparagine endopeptidase (AEP) cleavage products, tau N368 and α-synuclein N103 fragments, accompanied by a lower intracellular monoamine oxidase-B (MAO-B) activity. In the subacute MPTP-induced and chronic rotenone-induced PD mice models, we found Taltirelin (1 mg/kg) significantly improved the locomotor function and preserved dopaminergic neurons in the substantia nigra (SN). In accordance with the *in vitro* study, Taltirelin down-regulated the levels of p-tau (S396), p-α-synuclein (S129) tau N368 and α-synuclein N103 fragments in SN and striatum. Together, this study demonstrates that Taltirelin may exert neuroprotective effect *via* inhibiting MAO-B and reducing the oxidative stress and apoptosis, preventing AEP activation and its subsequent pathological cleavage of tau and α-synuclein, thus provides evidence for Taltirelin in protective treatment of PD.

## Introduction

Thyrotropin-releasing hormone (TRH) is widely known as a metabolism-regulating endocrine hormone acting through the hypothalamus-pituitary-thyroid axis (HPT axis). However, another role of TRH as a neuropeptide is underestimated (Gary et al., 2003; Khomane et al., 2011). TRH has long ago been proved useful in multiple animal models of diseases such as brain/spinal cord injuries (Faden et al., 1981; Pitts et al., 1995), depression (Szuba et al., 1995; Marangell et al., 1997), epilepsy (Jaworska-Feil et al., 2001; Kubek et al., 2009), schizophrenia (Wilson et al., 1973), amyotrophic lateral sclerosis (ALS; Hawley et al., 1987; Brooks, 1989), Alzheimer’s disease (AD; Mellow et al., 1989; Molchan et al., 1990) and Parkinson’s disease (PD; Ogata et al., 1998). However, TRH has the intrinsic shortcomings such as short half-life, poor lipophilicity and strong HPT axis stimulating effect, which severely restrict its application (Kinoshita et al., 1998). Taltirelin (TA-0910, Ceredist^®^), an oral-effective TRH analog, has 10–100 times more potent central nervous system (CNS) stimulant activity and eight times longer duration than TRH. More importantly, Taltirelin has been approved in the treatment of spinocerebellar degeneration (SCD), thus is highly promising in investigating other possible applications of TRH (Kinoshita et al., 1994, 1998).

PD is a neurodegenerative disease with the second high incidence among elderly over the age of 65 (Lee and Gilbert, 2016). Motor (such as bradykinesia, tremor and rigidity) and non-motor (such as depression and constipation) symptoms of PD are directly related to the significant loss of dopaminergic neurons in the substantia nigra (SN) and accordingly dopamine (DA) deficiency within the CNS. Although these symptoms are managed by pharmacological DA substitution, lack of the disease-modifying interventions, which could prevent further neuron loss thus slow or halt the progression of the disease, PD remains an incurable disease that causes severe disability and high mortality (Poewe et al., 2017). The death of dopaminergic neurons are caused by several factors, the most prominent one among which is the pathologically elevated intracellular α-synuclein, whose toxic forms (oligomers or fibrils) or deposition induces and exacerbates other neurotoxic events, such as mitochondrial function, oxidative stress and neuroinflammation, ultimately driving the neuron death in PD (Kalia and Lang, 2015). Nevertheless, growing evidence showed that α-synuclein and another pathologic protein tau could form hybrid oligomers and enhance their aggregation in the brains of PD and AD patients (Sengupta et al., 2015). The overlap of these proteinopathies not only emphasizes a similar structural characteristics of α-synuclein and tau (Moussaud et al., 2014), but also suggests the existence of a common upstream pathological trigger. Asparagine endopeptidase (AEP, or legumain), a pH-dependent endo-lysosomal cysteine protease, is found abnormally up-regulated in AD and PD. Once activated, AEP is able to cleave amyloid precursor protein (APP), tau and α-synuclein, whose protein fragments are more susceptible to hyperphosphorylation and aggregation, thus resulting in neurotoxicity (Zhang et al., 2014, 2016, 2017). Moreover, we have recently reported that the crosstalk among α-synuclein, monoamine oxidase-B (MAO-B), 3,4-dihydroxyphenylacetaldehyde (DOPAL) and AEP plays a central role in the pathogenesis of PD (Kang et al., 2018).

TRH and its analogs reveal neurotrophic and anti-apoptotic effects by counteracting the toxic effects of various cytotoxins in PD cellular models (Jaworska-Feil et al., 2010) *via* anti-apoptosis, inhibiting the activity of glycosynthase kinase-3β (GSK-3β), decreasing the phosphorylation level of tau (Luo and Stopa, 2004), protecting against Aβ toxicity and reducing the production of inflammatory cytokines TNF-α and IL-6 *in vitro* (Faden et al., 2005). However, the underlying mechanisms were still unclear. In this study, we showed that Taltirelin significantly reduced the toxicity of MPP+ and rotenone in SH-SY5Y cells and rat primary SN neurons, ameliorated the behavioral disturbance induced by MPTP or rotenone in PD mice *via* inhibiting MAO-B activity and reactive oxygen species (ROS) generation, reducing the abnormal phosphorylation of α-synuclein and tau, inhibiting the activation of AEP and its specific cleavage effect, thus provides evidence for Taltirelin in protective treatment of PD.

## Materials and Methods

### Animals

Male C57/BL6 mice, 8–9 weeks old (Beijing HFK Bioscience Co., Ltd., Beijing, China) weighing 20–25 g were used in this experiment. The animals were housed with food and water provided *ad libitum*, 12 h light/dark cycle, constant temperature and humidity. This study was carried out in accordance with the recommendations of the Guidelines of Laboratory Animals Ethics of Tongji Medical College, Huazhong University of Science and Technology. The protocol was approved by the Ethics Committee of Huazhong University of Science and Technology.

### Cell Culture

#### SH-SY5Y Neuroblastoma Cells Culture

SH-SY5Y neuroblastoma cells (Cell Resources Center of Shanghai Institutes for Biological Sciences) were grown in DMEM/F12 medium (Hyclone, South Logan, UT, USA) supplemented with 10% FBS (Gibco, Carlsbad, CA, USA) and 100 units/ml of penicillin and streptomycin. Cultures were maintained in a humidified incubator at 37°C/5% CO_2_ with medium changed every 3 days. After reaching 80% confluence, cells were seeded onto appropriate multi-well plates at a density of 4 × 10^5^ cells/ml.

#### Primary Neonatal Rat Midbrain Neurons Culture

Primary rat midbrain cultures were prepared as described previously (Beaudoin et al., 2012). In brief, the midbrain was dissected from P1–3 mouse pups. The isolated tissues were then chemically and mechanically dissociated into single cell suspensions. Cells were plated into 0.5 mg/ml PLL coated 6-well plates or glass coverslips at a density of 5.5–6 × 10^5^ per well. Incubate the cells with plating medium (DMEM/F-12 medium with 20% FBS, 100 units/ml of penicillin and streptomycin) for 4 h and replace the medium with maintenance medium: Neurobasal medium (Gibco, Carlsbad, CA, USA) with 2% B-27 supplement (Gibco, Carlsbad, CA, USA), 2 mM glutamine (Gibco, Carlsbad, CA, USA) and 100 units/ml of penicillin and streptomycin. Cultures were maintained in a humidified incubator at 37°C/5% CO_2_ for 3 days *in vitro* prior to functional studies.

#### MTT Assay

Cell viability was quantified by colorimetric assay with MTT (Beyotime, China) according to the manufacturer’s protocol. Briefly, 20 μl MTT (5 mg/ml) was added to each well (100 μl medium each) and incubated for 3 h at 37°C, then the dye was solubilized by 150 μl DMSO and the absorbance of each sample was measured at 570 nm with 630 nm as reference in a microplate reader (BioTek, Winooski, VT, USA). Cell viability = [(drug-treated well OD − MTT-free well OD)/(vehicle-treated well OD − MTT-free well OD)] × 100%.

#### Measurement of Lactate Dehydrogenase (LDH) Release

In order to estimate cell death, the level of lactate dehydrogenase (LDH) released from damaged cells into the culture media was measured after intervention. 1 h before the incubation endpoint, LDH release reagent was added to intervention-free wells as the maximum enzyme activity control. Then culture supernatants were collected and incubated with the appropriate reagent mixture according to the supplier’s instructions (LDH Cytotoxicity Assay Kit, Beyotime, China) at room temperature for 30 min. Absorbance of each well was measured at a wavelength of 490 nm with 630 nm as reference. The absorbance of MTT-free controls was subtracted from each value; Cell death rate = [(drug-treated well OD − vehicle-treated control well OD)/(maximum enzyme activity control well OD − vehicle-treated control well OD)] × 100%.

#### Identification of Apoptotic Cells

In order to visually assess DNA fragmentation in SH-SY5Y cells, Hoechst 33342 staining was applied after intervention. The cultures were washed with PBS and fixed for 20 min with 4% paraformaldehyde (PFA). After fixation, cultures were washed and exposed to Hoechst 33342 (5 μg/ml; Beyotime, Shanghai, China) for 15 min. After washing, coverslips were mounted on slides and images were recorded using inverted fluorescence microscope (Olympus, Japan) with excitation wavelength 350 nm (blue fluorescence). Cells with bright blue fragmented nuclei showing condensation of chromatin were identified as apoptotic cells and counted.

### PD Mice Model

#### Subacute MPTP-Induced PD Mice Model and Intervention

Mice were randomly divided into five groups: normal, MPTP, Taltirelin 0.2 mg/kg, 1 mg/kg, 5 mg/kg (*N* = 8). The intervention (14 days) was divided into two sections (7 days each). In the first section (MPTP + drug intervention), MPTP group was injected i.p. with MPTP (Sigma, St. Louis, MO, USA) of 30 mg/kg/d and three Taltirelin groups were pre-treated with Taltirelin (i.p.) of respective doses 4 h before MPTP injection for 7 days. In the second section (drug intervention), the MPTP group was given saline (i.p.), while three Taltirelin groups continued Taltirelin administration for another 7 days. Behavioral assessments and weights measurement were conducted on day 1, day 7 and day 14 of intervention period.

#### Chronic Rotenone-Induced Mice Model and Intervention

Mice were randomly divided into four groups: normal, rotenone, Taltirelin 0.2 mg/kg, 1 mg/kg (*N* = 8). Rotenone (4% Tween-20, 0.5% CMC; Sigma, St. Louis, MO, USA) was administered orally once a day at a dose of 30 mg/kg for 56 days. The Taltirelin groups were given Taltirelin i.p. of respective doses before gavage. Behavioral assessments and weights measurement were conducted on day 1 and day 56 of the intervention period.

### Behavioral Assessment

#### Rotarod Test

Mice were placed on a rotarod system (YLS-4C, Jinan Yiyan Technology Development Co., Ltd., China) that accelerated from 5 rpm to 40 rpm over a period of 90 s and 40 rpm was maintained for maximal 300 s. The length of time that animal was able to stay on the rod (latency time) was recorded. Test of each animal was repeated three times with at least 30 min interval between each experiment and the longest latency time was adopted. Before the formal experiments, adaptational trainings were conducted for 3 days and the animals were evenly divided into different groups based on their performances.

#### Pole Test

Mice in each group were placed head-up on the top of a vertical pole (50 cm tall and 1 cm in diameter). The base of the pole was placed in the home cage. The mice would turn its head downward and climb along the pole to the home cage. Time of the mouse head orient downward (time-turn) and total time from the top of the pole to home cage (time-down) as an indication of the locomotion activity was recorded. Each mouse received three successive trials and the average time was calculated. Before the formal experiments, mice in each group received training for 3 days.

#### Serum Thyroid Hormone Measurement

Blood was collected at 2 h post-administration of drugs and placed in 4°C overnight. Then the blood was centrifugated at 3,000 rpm for 15 min and serum was collected for further analysis. The levels of thyroid stimulating hormone (TSH), free and total thyroxine (T4) or triiodothyronine (T3) were measured using ELISA kit respectively (ALPCO) according to the manufacturer’s protocol. Briefly, 50 μL of serum samples or reference was pipetted into the assigned wells, followed by T3/T4 enzyme-conjugated solution with 1 h of incubation. The mixture was removed, and the plate was washed several times with water; then, working substrate solution was added to each well for reaction, and after 20 min in the dark, the reaction was stopped by adding 3 M HCl. Absorbance was read at 450 nm. The corresponding concentrations were calculated based on the standard curve.

#### Western Blot Analysis

For animal brain tissues, striatum or midbrain was dissected and homogenized with micro-tissue-grinders (Tiangen, OSE-Y30) in ice-cold enhanced RIPA lysis buffer [50 mM Tris, pH 7.4, 150 mM NaCl, 1% Triton X-100, 1% sodium deoxycholate, 0.1% SDS, 5 mM EDTA, 2 mM Na_3_VO_4_, 1 mM PMSF, 10 mM NaF and a cocktail of protease inhibitors (Roche, Branchburg, NJ, USA)]. For cellular culture, cells were scraped down by a cell scraper and lysed in lysis buffer. Brain or cellular lysates were centrifuged at 12,000 *g* at 4°C for 15 min and the protein concentrations were determined by a BCA assay kit (Pierce, Rockford). The supernatants were boiled for 10 min with 1% SDS loading buffer. After SDS-PAGE, the samples were transferred to a PVDF membrane (Millipore, USA), blocked with 5% non-fat milk for 1 h at room temperature and incubated overnight at 4°C with primary antibodies against following proteins: tyrosine hydroxylase (TH; 1:1,000, Proteintech, 25859-1-AP), p44/42 MAPK (ERK1/2; 1:2,000, Cell Signaling, #4695), phospho-p44/42 MAPK (ERK1/2; 1:2,000, Cell Signaling, #4370), α-synuclein (1:1,000, Abcam, ab85862), phospho-α-synuclein (1:1,000, Cell Signaling, #23706), phospho-tau (S396; 1:10,000, Abcam, ab109390), cleaved Caspase-3 (1:1,000, Cell Signaling, #9661), GAPDH (1:2,000, AntGene, ANT012), tau N368 and α-synuclein N103 (1:1,000, provided by Professor Keqiang Ye of Emory University). The membranes were washed with TBST, and then incubated with HRP-conjugated anti-rabbit secondary antibody (1:3,000, Antgene, ANT020) for 1 h at room temperature. After washing, the membrane was visualized with an ECL detection kit (Thermo Scientific) in the Bio-Rad imaging system. Bands intensities were analyzed with ImagineJ software.

### Immunohistochemistry and Immunofluorescent Staining

#### Immunohistochemistry Staining

Animals were deeply anesthetized and transcardially perfused with saline, followed by 4% PFA in 0.1 M PBS (pH 7.4). Brain were dissected and fixed in 4% PFA overnight at 4°C. After fixation, tissues were transferred to 70% ethanol and processed for paraffin sectioning (4 μm/section). The sections were mounted on glass slides, deparaffinized by xylene, dehydrated in graded ethanol solutions, baked in the basic antigen retrieval buffer (pH = 6.0) and washed with phosphate buffer (pH 7.4). After washing, sections were blocked with 3% bovine serum albumin (BSA) and then incubated with diluted primary antibody in a humidified chamber overnight at 4°C. Primary antibodies against following proteins were used: TH (1:1,000, Proteintech, 25859-1-AP), phospho-tau (S396; 1:10,000, Abcam, ab109390). The sections were washed and subsequently incubated with biotinylated goat anti-rabbit IgG, then HRP labeled streptavidin fluid, followed by DAB solutions, counterstained with Harris hematoxylin, dehydrated in graded ethanol solutions and eventually cover slipped. Images were collected through an Olympus camera connected to the microscope at the same light intensity, and analyzed using Image-Pro Plus software.

#### Immunofluorescent Staining

For brain slices, immunofluorescent staining shared a same procedure with immunohistochemistry staining before secondary antibodies incubation. For cellular cultures, sterile coverslips were placed in 24-well plates and coated with PLL before cells were plated on the coverslips. After intervention, cells were first washed with PBS and fixed with 4% PFA. Then cells were permeabilized with 0.2% Triton X-100 and blocked with 5% BSA. Incubate brain slices or cell slides with following primary antibodies overnight at 4°C: α-synuclein (1:1,000, Abcam, ab85862), tau N368 (1:1,000, provided by Professor Keqiang Ye of Emory University). After being washed, sections were incubated in dark with appropriately diluted Alexa 488- or Cyanine 3-coupled secondary antibodies followed by DAPI staining nucleus for 10 min. To stain nuclei, incubate with DAPI for 10 min. Coverslips were mounted on slides with one drop per coverslip of antifade mounting medium (Beyotime, China). Images were collected using fluorescence microscopy with image manipulation software, and analyzed using Image-Pro Plus software.

### Flow Cytometric Analysis of Reactive Oxygen Species (ROS)

After pre-treatment of PBS or Taltirelin (5 μM) for 0.5 h, SH-SY5Y cells were incubated with MPP+ (1,000 μM) or rotenone (50 μM) for 24 h. Then cells were stained with 2′,7′-dichlorodihydrofluorescein diacetate (DCFH-DA; Beyotime, China) according to the protocol. Briefly, the culture medium was removed and cells were washed twice with serum-free culture medium. Next, the cells were loaded with DCFH-DA (10 μM) in serum-free culture medium for 20 min in 37°C. After being washed twice with PBS, cells were digested with trypsin, then washed once with PBS and finally collected. The samples were analyzed for the fluorescence (488 nm excitation and 525 nm emission) of DCF by flow cytometry (FACS LSR Fortessa System, BD Biosciences, San Jose, CA, USA).

### Monoamine Oxidase-B (MAO-B) Activity Assay

After pre-treatment of PBS or Taltirelin (5 μM) for 0.5 h, SH-SY5Y cells were incubated with MPP+ (1,000 μM) or rotenone (50 μM) for 24 h. Next, cells were scraped down by a cell scraper and homogenized in PBS with cocktail of protease inhibitors. Large debris was then removed by weak centrifugation (1,000 *g*, 10 min). Next, the supernatant was collected and incubated with the working solution of 100 μl containing 400 μM Amplex Red reagent, 2 U/ml HRP and 2 mM benzylamine substrate (Molecular Probes, Eugene, OR, USA). The fluorescence of MAO-B activity was measured in a fluorescence plate reader (530–560 nm excitation and 590 nm emission).

### Statistic Analysis

Data were analyzed using one-way analysis of variance (ANOVA) followed by Tukey HSD or LSD *post hoc* tests for multiple comparisons between groups, and Student’s *t*-test for comparing two groups. All statistical analyses were performed in SPSS 21.0 software. The significance level was set at *p* < 0.05. Data are presented as Mean ± SEM for all results.

## Results

### Taltirelin Protected Dopaminergic Neurons Against Apoptosis *in vitro*

**Figure 1:**
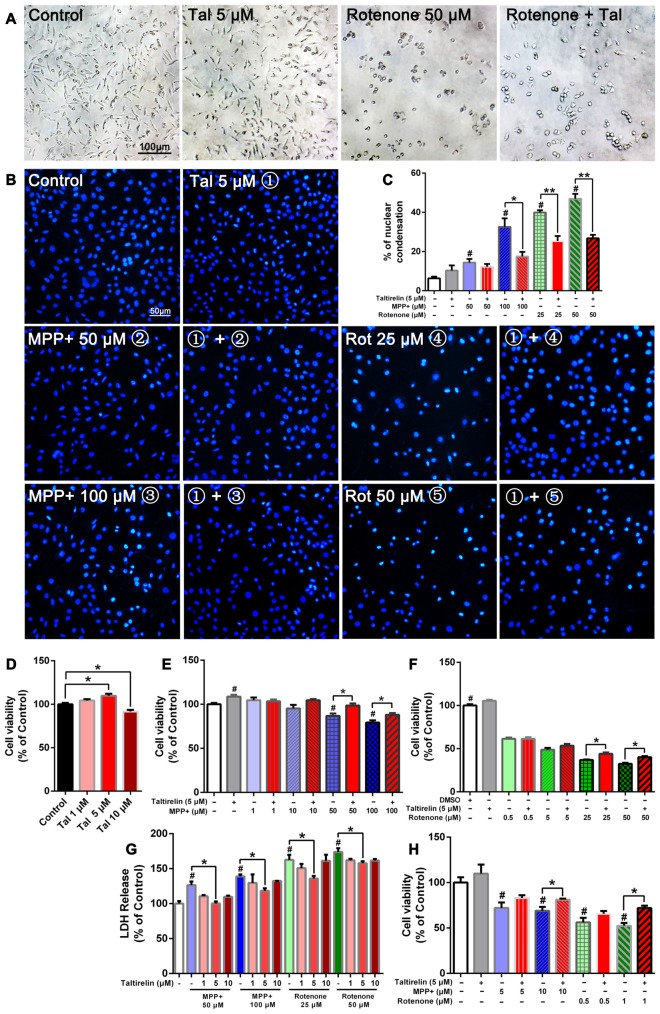
Taltirelin protected dopaminergic neurons from toxicity of MPP+ and rotenone. SH-SY5Y cells were pre-treated with Taltirelin (5 μM) for 2 h and incubated another 24 h with MPP+ (1, 10, 50 or 100 μM) or rotenone (0.5, 5, 25 or 100 μM; **(A)** phase contrast micrograph of cultured SH-SY5Y cells exposed to rotenone (50 μM) for 24 h. **(B)** Hoechst 33342 staining of SH-SY5Y cells. **(C)** The percentage of nuclear condensation in total cell population as measured by Hoechst 33342 staining.** (D–F)** SH-SY5Y Cell viability was measured by MTT assay after intervention. **(G)** Lactate dehydrogenase (LDH) release assay was used to determine the cell death rate of SH-SY5Y cells. **(H)** Viability of primary midbrain neurons was assessed by MTT assay. ^#^*p* < 0.05 vs. Control; *p < 0.05; **p < 0.01. *N* = 6. Error bars represent SEM.

The neuroblastoma SH-SY5Y cells are a well-established and widely-used *in vitro* model of dopaminergic neuron origin. We found 5 μM Taltirelin induced the highest proliferation (109.70 ± 2.41% of control, *p* < 0.05) of cells after 24-h-incubation, while higher concentration (10 μM) caused a decrease of cell viability (91.64 ± 1.90% of control, *p* < 0.05; Figure 1D). Thus, we adopted 5 μM for our following experiments.

Consistent with the previous studies, SH-SY5Y cells exposed to rotenone for 24 h underwent substantial reduction of viable cells and exhibited early apoptotic cell death morphology, such as cell shrinkage and disappearance of neurite processes (Figure 1A). In contrast, MPP+ caused the decrease of cell numbers, but morphological changes were not obvious (data not shown). Moreover, Hoechst 33342 staining showed that MPP+ (50 μM, 100 μM) and rotenone (25 μM, 50 μM) increased the number of bright staining nuclei which was due to chromatin condensation (14.37 ± 2.01%, 32.60 ± 4.33%, 39.91 ± 1.16%, 46.93 ± 2.49% respectively vs. 6.33 ± 0.88% of control, *p* < 0.05; Figure 1B). Consistently, we quantified the cell viability by MTT assay and LDH release assay. As expected, both MPP+ (50 μM, 100 μM) and rotenone (25 μM, 50 μM) induced a dose-dependent decrease of cell viability (86.76 ± 2.81%, 79.33 ± 2.42%; 36.96 ± 0.60%, 32.43 ± 1.20% of control, *p* < 0.05; Figures 1E,F) and increase of LDH release (126.39 ± 5.21%, 138.99 ± 2.69%; 162.63 ± 7.09%, 173.95 ± 5.31% of control, *p* < 0.05), which indicated an increase of cell death (Figure 1G).

Pre-treatment of Taltirelin (5 μM) for 2 h significantly alleviated the injury caused by MPP+ or rotenone. Taltirelin decreased nuclear condensation by 46.37% (100 μM MPP+), 36.52% and 42.99% (25 μM and 50 μM rotenone, respectively) and recovered cell viability by 13.58% and 11.06% (50 μM and 100 μM MPP+ respectively), 19.26% and 23.78% (25 μM and 50 μM rotenone respectively; Figures 1A–G). Besides, we conducted similar experiments using primary midbrain neurons and observed a similar viability-improving effect of Taltirelin against 10 μM MPP+ (81.17 ± 1.10% vs. 68.99 ± 4.26% of control, *p* < 0.05) and 1 μM rotenone (52.45 ± 3.06% vs. 71.93 ± 2.64% of control, *p* < 0.05; Figures 1A–H), which further confirmed the protective effect of Taltirelin in dopaminergic neurons *in vitro*.

### Taltirelin Down-Regulated Tau and α-Synuclein-Related Pathology *in vitro*

**Figure 2:**
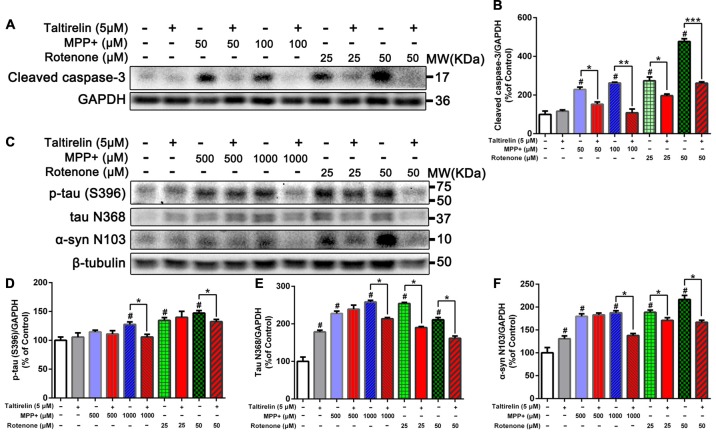
Taltirelin down-regulating tau and α-synuclein-related pathology *in vitro*. **(A)** Western blot analysis showed the expression of cleaved caspase-3 in SH-SY5Y cells exposed to MPP+ (50 or 100 μM) or rotenone (25 or 50 μM), with or without 2 h pre-treatment of Taltirelin (5 μM). **(B)** Quantification of cleaved caspase-3 in different groups. **(C)** Western blot analysis showed the expression of p-tau (S396), tau N368 and α-synuclein N103 in SH-SY5Y cells exposed to MPP+ (500 or 1,000 μM) or rotenone (25 or 50 μM), with or without 2 h pre-treatment of Taltirelin (5 μM). **(D–F)** Analysis of p-tau (S396), tau N368 and α-synuclein N103 vs. β-tubulin in different groups. ^#^*p* < 0.05 vs. control; *p < 0.05, **p < 0.01, ***p < 0.001. *N* = 3. Error bars represent SEM.

After being cleaved by the upstream caspase, caspase-3 is activated and execute apoptosis by further cleaving targeted cellular proteins, thus the level of cleaved caspase-3 is positively correlated with the extent of apoptosis (Elmore, 2007). We found that the level of cleaved caspase-3 in SH-SY5Y cells was significantly elevated by MPP+ (50 μM, 100 μM) and rotenone (25 μM, 50 μM). However, Taltirelin (5 μM) pre-incubation effectively prevented the elevation of the cleaved caspase-3 (decreased by 33.41%, 58.62%, 28.11% and 45.23% respectively, *p* < 0.05; Figures 2A,B), which further confirmed that Taltirelin protected dopaminergic neurons against apoptosis and preserved the cell viability.

Since α-synuclein of human but not rodents contains residue N103 thus the α-synuclein N103 fragment is selectively cleaved by AEP (Zhang et al., 2017), we further examined the impact of Taltirelin on tau and α-synuclein pathologies in SH-SY5Y cells of human origin. As expected, MPP+ (1,000 μM) and rotenone (25 μM, 50 μM) significantly increased the levels of p-tau (S396) and AEP cleavage products: tau N368 and α-synuclein N103 fragments. Taltirelin (5 μM) co-incubation reduced p-tau (S396) by 17.13% (1,000 μM MPP+) and 10.12% (50 μM rotenone), tau N368 by 17.28% (1,000 μM MPP+), 25.22% (25 μM rotenone) and 23.40% (50 μM rotenone), α-synuclein N103 by 26.55% (1,000 μM MPP+), 9.49% (25 μM rotenone) and 23.01% (50 μM rotenone; Figures 2C–F, *p* < 0.05). Thus, these results suggested that Taltirelin had a neuroprotective effect involved reducing the phosphorylation of tau and inhibiting the activation of AEP induced by MPP+ and rotenone.

### Taltirelin Protected Locomotor Functions of PD Model Mice

**Figure 3:**
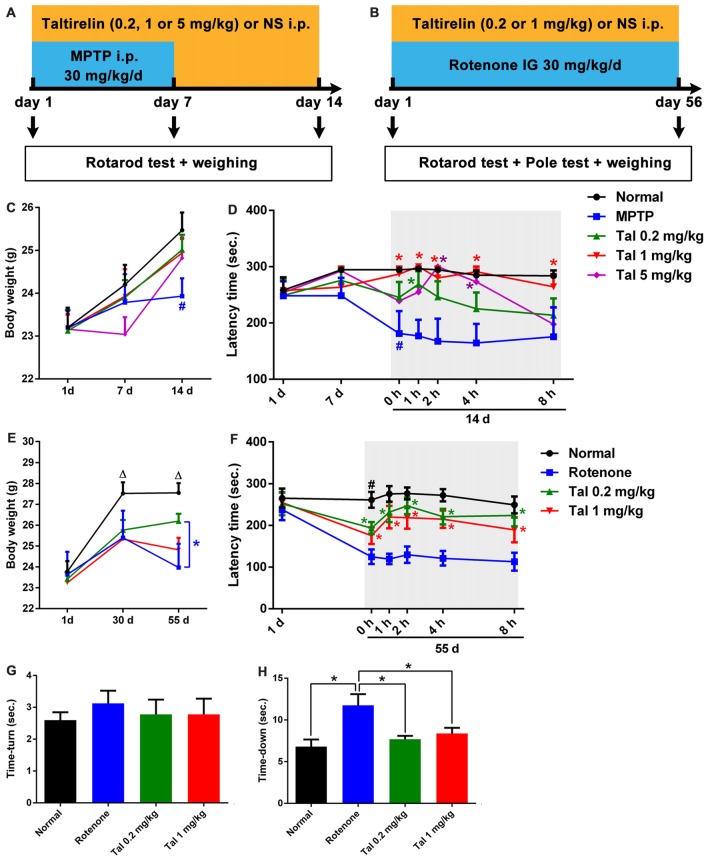
Taltirelin protected locomotor functions of Parkinson’s disease (PD) model mice. Schematic representation of the subacute MPTP-induced **(A)** and the chronic rotenone-induced **(B)** PD mice model experimental design. **(C)** Weight measurements on the first, 7th and 14th day of mice with MPTP (30 mg/kg, i.p.), with or without Taltirelin (Tal 0.2, 1 and 5 mg/kg). **(D)** Rotarod tests on the first, 7th and 14th day of MPTP modeling. **(E)** Weight measurements on the first, 30th and 55th day of mice with rotenone (30 mg/kg, IG), with or without Taltirelin (0.2 and 1 mg/kg). **(F)** Rotarod tests on the first and 55th day of rotenone modeling. **(G,H)** Pole tests on the 55th day of rotenone modeling. ^#^*p* < 0.05 vs. normal; *p < 0.05; ^△^*p* < 0.05 vs. other groups. *N* = 4. Error bars represent SEM.

We further assessed the neuroprotective effects of Taltirelin in the subacute MPTP-induced PD mice model (Figure 3A). In the pilot test, we found that the mice injected with MPTP (30 mg/kg, i.p.) 2 h after Taltirelin (5 mg/kg, i.p.) would develop irritation, spasm and finally death, which may be due to the metabolism increased by Taltirelin aggravated the toxicity of MPTP. Therefore, the injection interval was adjusted to 4 h and similar death was prevented. Possibly due to the same reason, after 7 days of intervention with MPTP, the body weights of the mice injected with 5 mg/kg Taltirelin (23.04 ± 0.90 g) was lower than other groups (24.20 ± 1.31 g), but gradually recovered (25.47 ± 1.09 g) after following Taltirelin protective treatment (7 days), while the body weight of the MPTP group (23.43 ± 0.83 g), obviously decreased on the 14th day. However, there was no significance difference between MPTP and Taltirelin groups (Figure 3C). On the 7th day of the MPTP modeling, no obvious performance differences in the rotarod test were observed among groups, but on the 14th day, the latency times in the rotarod test in the MPTP group (179.33 ± 94.62 s, *p* < 0.05) significantly decreased, indicating impaired locomotor functions. Taltirelin 1 mg/kg (287.20 ± 28.62 s) successfully alleviated the impairment induced by MPTP. Besides, there was a trend of better performance of Taltirelin 0.5 mg/kg group (245.5 ± 77.25 s) or 5 mg/kg (239.20 ± 75.31 s) group compared with MPTP group (Figure 3D).

We also examined the impacts of Taltirelin in rotenone-induced chronic (Figure 3B) neurotoxicity mice models. After the chronic modeling (rotenone, 30 mg/kg, IG, 55 days), the weights of mice declined with different degrees compared with the normal mice. The mice injected with 1 mg/kg Taltirelin (26.20 ± 1.04 g vs. 23.03 ± 1.53 g of MPTP group, *p* < 0.05) had the least reduction (Figure 3E). In the rotarod test, mice of 0.2 mg/kg Taltirelin group (193.78 ± 45.66 s) and 1 mg/kg Taltirelin (182.92 ± 56.64 s) performed much better than the rotenone group (124.92 ± 49.63 s; Figure 3F, *p* < 0.05). In addition, the mice of rotenone group (11.75 ± 3.81 s) in the pole test exhibited much slower decline speed or even declined in a rotatory manner, a sign of significant motor impairment. Both 0.2 mg/kg (7.70 ± 1.25 s) and 1 mg/kg Taltirelin (8.38 ± 1.92 s) rescued the time-down of the mice (Figure 3G, *p* < 0.05). However, there was no obvious difference of time-turn among different groups (Figure 3H). Anyhow, the above behavior assessments suggested a neuroprotective effect of Taltirelin in MPTP or rotenone PD mice models.

In addition, we also examined the motor improving effect of Taltirelin immediately after injections and found a time- and dose-dependent locomotor improvement of both MPTP and rotenone mice, which could maintain for at least 6 h (*p* < 0.05). This result was in consistency with our findings that Taltirelin could improve motor function of 6-OHDA lesioned PD rats (unpublished data).

### Taltirelin Protected Dopaminergic Neurons and Reduced PD-Related Pathologies in MPTP-Induced PD Mice Model

**Figure 4:**
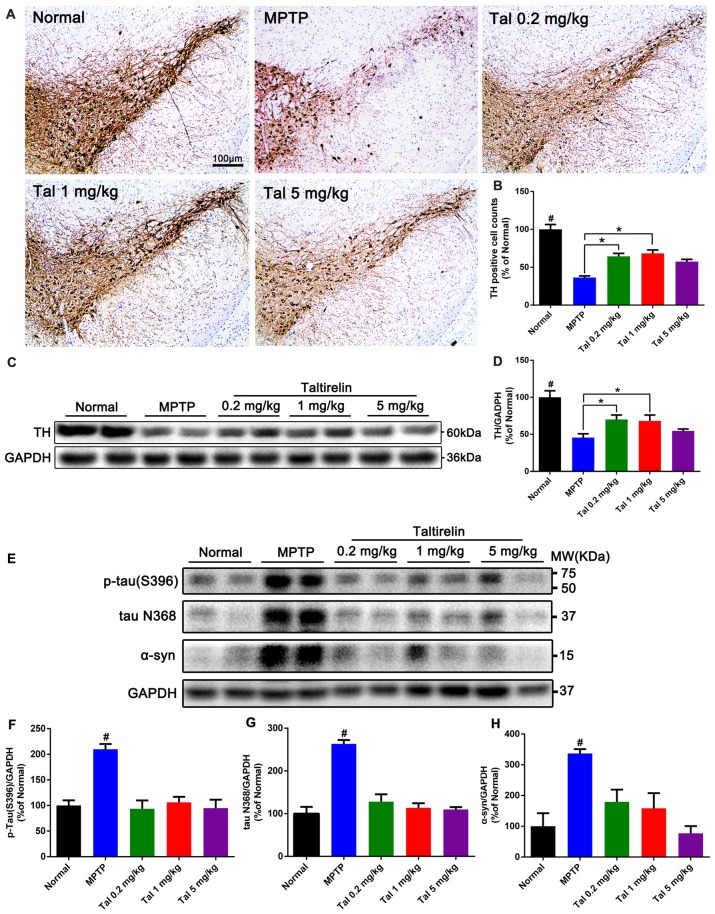
Taltirelin protected dopaminergic neurons and reduced PD-related pathologies in MPTP-induced PD mice model.** (A)** Tyrosine hydroxylase (TH) immunostaining of the substantia nigra (SN) of mice treated with MPTP (30 mg/kg, i.p.), with or without Taltirelin (0.2 and 1 mg/kg). **(B)** Analysis of TH positive cells counts in each group. **(C)** Western blot analysis showed the levels of TH in the striatum of each group. **(D)** Quantification of the levels of TH.** (E)** WB showed the levels of p-tau (S396), tau N368 and α-synuclein in the SN of each group. **(F–H)** Quantification of WB of p-tau (S396), tau N368 or α-synuclein vs. GAPDH. ^#^*p* < 0.05 vs. other groups; *p < 0.05. *N* = 3. Error bars represent SEM.

In order to quantify the protective effect of Taltirelin, we stained the brain slices of SN with TH, the rate-limiting enzyme in DA synthesis which mainly expressed by dopaminergic neurons. We found that the numbers of TH positive neurons were much fewer in MPTP treated mice (36.66 ± 2.08% of normal) than that in 0.2 mg/kg (64.47 ± 3.91%) or 1 mg/kg (68.29 ± 4.45%) Taltirelin groups (Figures 4A,B, *p* < 0.05). We also quantified the level of TH in the striatum using western blot and the results validated the immunostaining observation (Figures 4C,D, *p* < 0.05).

We further examined the level of toxic forms of tau and α-synuclein in the SN and found that compared with the normal group, the levels of p-tau (S396), tau N368 and α-synuclein of the MPTP group were significantly higher, whereas the levels of the above proteins were reduced by Taltirelin of three doses by at least 55.43% (Figures 4E–H). Therefore, consistently with *in vitro* results, Taltirelin may have a protective effect against MPTP-induced neurotoxicity on dopaminergic neurons by decreasing the phosphorylation of tau and α-synuclein and down-regulating activation of AEP.

### Taltirelin Down-Regulated Tau and α-Synuclein-Related Pathology in Rotenone-Induced PD Mice Model

**Figure 5:**
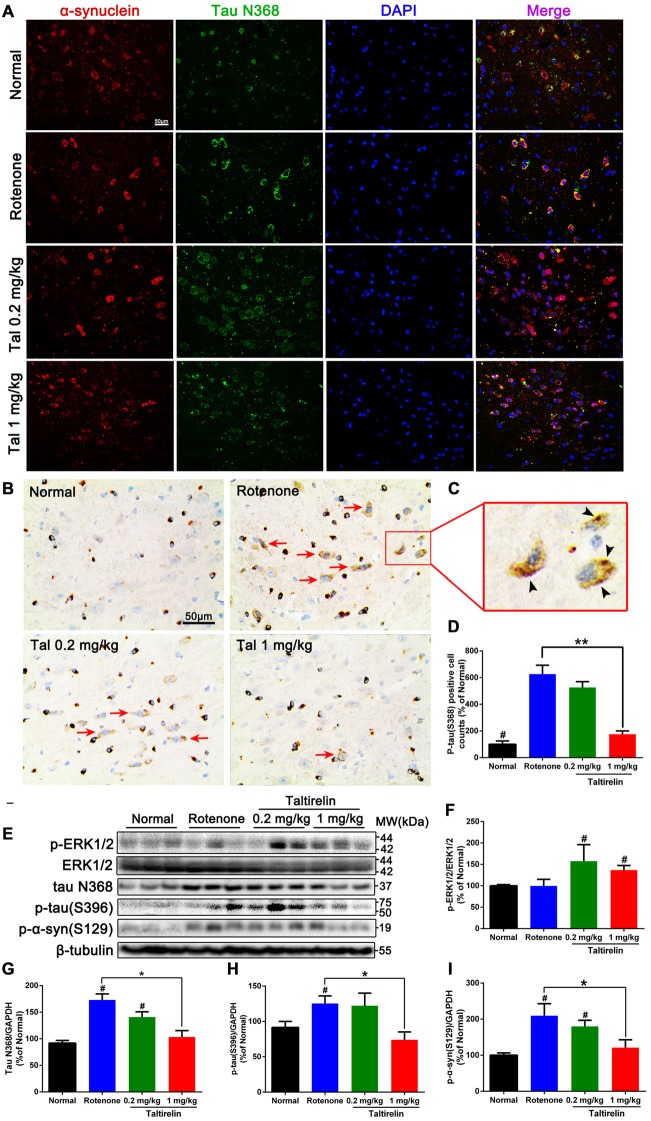
Taltirelin down-regulated tau and α-synuclein-related pathology in rotenone-induced PD model mice. **(A)** Double-labeled immunofluorescence images showed co-localization of α-synuclein and tau N368 in the SN of each group (normal, rotenone, 0.2 or 1 mg/kg Taltirelin).** (B)** Immunostaining of p-tau (S396) in the SN. Red arrows indicated the positive cells. **(C)** The enlarged graph of indicated field. Black arrowheads indicated intracellular p-tau (S396) aggregation. **(D)** Analysis of p-tau (S396) positive cells counts in each group. **(E)** Western blot analysis of p-ERK1/2, tau N368, p-tau (S396) and p-α-synuclein (S129) in the SN of each group.** (F–I)** Quantification of WB showed the levels of p-ERK1/2, tau N368, p-tau (S396) and p-α-synuclein (S129). ^#^*p* < 0.05 vs. other groups; *p < 0.05; **p < 0.01. *N* = 3. Error bars represent SEM.

Our previous studies have shown co-localization and increase of α-synuclein and tau N368 in the rotenone PD mice model (Nie et al., 2015). Thus, we also performed immunofluorescence double-labeling of the above two proteins in the SN, striatum, hippocampus and cortex of rotenone mice. The results showed that normal mice also had α-synuclein and tau N368 co-localized and evenly distributed within the neurons, whereas in the rotenone group, intracellular α-synuclein and co-localized tau N368 exhibited higher and denser fluorescence with wider distribution in the cytoplasm, indicating increased and aggregation of α-synuclein and tau N368. In contrast, Taltirelin, especially 1 mg/kg group had much fewer and more dispersive distribution of α-synuclein and tau N368 in the SN (Figure 5A) and striatum (Supplementary Figure S1). However, similar difference among groups in the expression of two proteins was not significant in the hippocampus and cortex (Supplementary Figures S2, S3). Besides, we also stained p-tau (S396) in the SN and found an elevation of the number of p-tau (S396) positive cells in the rotenone group (626.57 ± 66.31% of normal), in which immunostaining positive dense particles of aggregating p-tau (S396) could be easily spotted. In the 1 mg/kg Taltirelin group (175.44 ± 25.06%), both the number of p-tau (S396) positive cells and intracellular dense particles significantly decreased (Figures 5B–D, *p* < 0.01).

Similarly, we also examined the levels of p-ERK1/2 and tau N368, p-tau (S396), p-α-synuclein (S129) in the SN. Compared with the rotenone group, the levels of tau N368 (103.50 ± 11.86% vs. 173.10 ± 11.39%, *p* < 0.05), p-tau (S396; 74.01 ± 11.24% vs. 125.40 ± 10.71%, *p* < 0.05) and p-α-synuclein (S129; 121.20 ± 21.55% vs. 209.60 ± 33.19%, *p* < 0.05) were significantly lower in the 1 mg/kg Taltirelin group (Figures 5E–I), which further confirmed the immunostaining results above. In our previous study, we observed a rise of p-ERK1/2 after Taltirelin treatment both *in vivo* and *in vitro* (unpublished data). Consistently, results showed that p-ERK1/2 increased in the SN and striatum of the 0.2 mg/kg (157.50 ± 38.55%, *p* < 0.05) and 1 mg/kg (136.8 ± 10.67%, *p* < 0.05) Taltirelin group, suggesting the involvement of p-ERK1/2 signaling in the actions of Taltirelin. Above all, the findings with rotenone mice further supported the observations in MPTP mice, indicating that Taltirelin has a neuroprotective effect associated with down-regulating AEP-related pathologies.

### Taltirelin Reduced the Generation of ROS and the Activity of MAO-B

**Figure 6:**
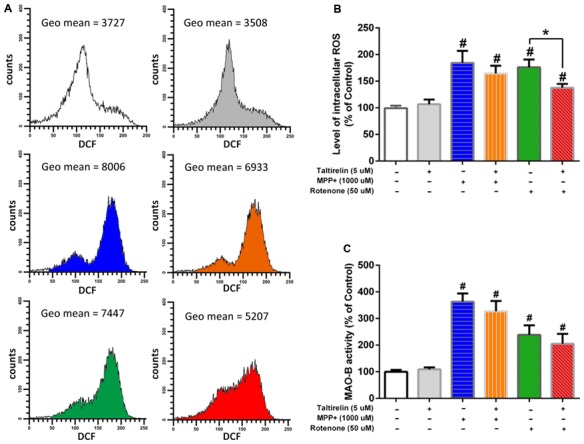
Flow cytometry analysis of reactive oxygen species (ROS) generation and monoamine oxidase-B (MAO-B enzymatic assay. SH-SY5Y cells were pre-treated with PBS or Taltirelin (5 μM, 2 h), then incubated with MPP+ (1,000 μM, 24 h) or rotenone (50 μM, 24 h).** (A)** One typical experiments of Flow cytometry analysis. Geometric mean (Geo Mean) was used to calculate the total intensity of 2′,7′-dichlorofluorescein (DCF) fluorescence of cells.** (B)** Quantitative analysis of DCF fluorescence intensity in each treatment group. **(C)** MAO-B activity was assessed by Amplex^®^ red monoamine oxidase assay kit. ^#^*p* < 0.05 vs. control; *p < 0.05. *N* = 3. Error bars represent SEM.

It is reported that oxidative stress facilitates accumulation (Conway et al., 2001) and oligomerization (Norris et al., 2003) of α-synuclein, which could be promoted by age-dependent activation of MAO-B (Kang et al., 2018). Thus, in order to get a further insight of the protective mechanisms of Taltirelin, we assessed the influence of Taltirelin on the ROS generation and MAO-B activity. Flow cytometry analysis showed that SH-SY5Y cells treated with MPP+ (1,000 μM) or rotenone (50 μM) for 24 h exhibited higher intracellular ROS (184.36 ± 22.63%, 176.16 ± 14.66%, respectively, *p* < 0.05). Pre-treatment with Taltirelin (5 μM) for 2 h significantly lowered the ROS elevated by rotenone by 38.65% ± 7.28% (*p* < 0.05) and decreased ROS induced by MPP+ by 19.83 ± 8.02%, though of no significance (Figures 6A,B). MPP+ (1,000 μM) and rotenone (50 μM) induced obvious increase of MAO-B activity (363 ± 30.58%, 238.41 ± 35.97%, respectively, *p* < 0.05) in SH-SY5Y cells. Cells pre-treated with Taltirelin (5 μM) exhibited a not significant but consistent decrease of the MAO-B activity elevated by MPP+ (by 36.15 ± 10.79%, *p* > 0.05) and rotenone (by 33.02 ± 6.71%, *p* > 0.05) (Figure 6C). Thus, these results suggested that Taltirelin may alleviate the neurotoxicity of MPTP and rotenone *via* reducing the generation of ROS and decreasing the activity of MAO-B.

## Discussion

TRH is stored in the releasable pool in the synaptic terminals and widely distributed in anatomically distinct pathways throughout the neuroaxis (Gary et al., 2003), regulating a variety of physiological activities in the nervous system. The neuroprotective effects of TRH and its analogs have been showed in animal models of cerebral ischemia (Urayama et al., 2002), cellular models of PD (Jaworska-Feil et al., 2010) and AD (Luo and Stopa, 2004). Here, we report that Taltirelin, a TRH analog had neuroprotective effect in both cellular and animal models of PD.

MPP+ (metabolite of MPTP) and rotenone, highly lipophilic insecticide, both are mitochondrial complex I inhibitors which impair the mitochondrial respiration chain, increase ROS production and trigger apoptosis of dopaminergic neurons (Giordano et al., 2012; Xicoy et al., 2017). MTT assay and the LDH release assay showed that Taltirelin (5 μM) co-incubation for 24 h alleviated cytotoxicity induced by MPP+ or rotenone and rescued the viability of both SH-SY5Y cells and primary midbrain neurons. Hoechst 33342 staining and the level of cleaved caspase-3 further suggested that Taltirelin reduced the apoptosis caused by MPP+ and rotenone. Activation of ERK1/2 is found to be able to initiate a series of anti-apoptosis processes (Roskoski, 2012). We have demonstrated that the actions of Taltirelin both *in vitro* and *in vivo* were companied by an elevation of p-ERK1/2, which may mediate the resistance to apoptosis of Taltirelin. We further established the subacute MPTP-induced and chronic rotenone-induced PD mice models and found that Taltirelin significantly rescued the locomotor functions of these animals. However, long-term and high-dose administration of Taltirelin led to the loss of weight in animals, which is most likely due to an over-activation of HPT axis. We also measured the level of TSH and thyroid hormones in the serum of mice and found a significant increase of total T4 when the dose of Taltirelin exceeded 0.2 mg/kg, while free T4 or T3 had no significant alteration (Supplementary Figure S4). Since the total T4 is of the highest concentration among thyroid hormones and also the effector hormones, the results suggested that the thyroid function of patients should be thoroughly evaluated and adjusted accordingly before administration of Taltirelin if possible, in the future.

AEP, also known as legumain, is an endo-lysosomal cysteine protease activated under acidic conditions. The pH within the brain decreased with aging, while pH in the brains of patients of AD or PD is generally low and AEP is significantly elevated (Basurto-Islas et al., 2013). The cleaved products of AEP, tau N368 or α-synuclein N103 fragments are themselves highly neurotoxic and are prone to hyperphosphorylation and aggregation. We thus examined the influence of Taltirelin on these AEP-related pathologies both *in vitro* and *in vivo*. MPTP and rotenone caused an elevation of p-tau (S396), p-α-synuclein (S129), tau N368 and α-synuclein N103 fragments in SH-SY5Y cells and in the SN of PD mouse models, while Taltirelin successfully reduced the levels and aggregation of tau or α-synuclein in toxic forms. We recently reported that MPP+ could increase endogenous MAO-B, α-synuclein and AEP protein levels in SH-SY5Y cells in a time-dependent manner (Kang et al., 2018), while MAO-B activation is closely related with increase in free radical damage and ROS (Kang et al., 2018). In addition, rotenone administration may interfere with DA distribution and metabolism, leading to DA accumulated in the cytoplasm of PC12 cells, which may contribute to the ROS formation and cell death (Sai et al., 2008). Our results showed that Taltirelin decreased the generation of ROS induced by rotenone in SH-SY5Y cells though no significance was with MPP+. Taltirelin also moderately reduced activity of MAO-B, which was elevated by MPP+ and rotenone though the reduction was not significant. As for possible explanations for the insignificance: First, MAO-B mainly exists in glial cells, while SH-SY5Y cells as a neuron-origin species may contain low expression levels of MAO-B (Maruyama et al., 2003) to demonstrate the influence of Taltirelin. Second, previous studies showed that the amount of DA that SH-SY5Y cells could synthesize and release is barely detectable (Xicoy et al., 2017). However, MAO-B-induced DA metabolite, DOPAL, is neurotoxic *per se* and is able to activate and raise protein levels of both AEP and MAO-B (Kang et al., 2018). Since Taltirelin is a potent and sustained DA-releasing agent, which decreases the intracellular DA, this part of contribution to reducing MAO-B activity could not be elucidated under this condition. Thus, it is predictable that *in vivo* assessment of MAO-B activity or elevating MAO-B level with genetic manipulations could provide more insight into the mechanisms.

Besides, other possible explanations for the protective mechanism of Taltirelin may exist. First, Taltirelin mainly interacts with G-protein coupled receptors, TRH type 1 receptor (TRH-R1). As with many other rhodopsin-like GPCRs, the mitogen-activated protein kinase (MAPK; or Ras-Raf-MEK-ERK1/2) pathway has been found to be a downstream effector of TRH-R1 signaling (Smith et al., 2001; Oride et al., 2008). We also observed that Taltirelin caused a rise of p-ERK1/2 in the striatum and SN of mice after protective treatment. p-ERK1/2 participates in many signaling pathways and may therefore mediate the protective effects of Taltirelin. Second, TRH and its analogs are able to increase cerebral blood flow and metabolism which often decline with aging and in PD or AD (Farkas et al., 2000; Borghammer et al., 2010; Austin et al., 2011), thus achieve the protection by accelerating the elimination of toxins in the CNS and reducing the formation of anoxia or toxins-induced acidic micro-environment, which is a trigger in AEP activation. Finally, multiple studies have suggested that TRH acted as a regulator of homeostasis (Gary et al., 2003) involving immune and endocrine system, whereas imbalance of inflammation (Zheng et al., 2016) or diabetes (Arvanitakis et al., 2004) plays a recognized role in neurodegenerative diseases like PD or AD. Therefore, it will be interesting to investigate the interaction of TRH or Taltirelin with internal environment in PD or AD.

It is widely accepted that thyroid function has great impact on cognition. Deficiency of thyroid hormone in the critical period of brain development leads to severely stunted physical and mental growth, such as Cretinism. No surprise, functions of the HPT axis have been shown to undergo numerous changes with aging (Roelfsema and Veldhuis, 2013). Although there are still many controversies regarding the relationship between thyroid function and neurodegenerative disease (Villanueva et al., 2013), study showed that the free T3 level was significantly lower in PD patients especially with akinetic-rigid motor subtype, while TSH level was lower in patients with tremor-dominant type or mixed type, suggesting a relation of the thyroid hormone level with PD motor symptoms (Umehara et al., 2015). TRH is decreased in the hippocampus of AD patients (Luo et al., 2002) and alterations in TSH (van Osch et al., 2004) or thyrotropin (Tan et al., 2008) have also been implicated as a risk factor for AD and dementia. Rat models of hypothyroidism exhibited increases of APP protein (Contreras-Jurado and Pascual, 2012), tau hyperphosphorylation, proinflammatory cytokines and spatial memory impairments (Chaalal et al., 2014), indicating that hypothyroidism represents an important risk factor of developing sporadic forms of AD. Since the regulatory functions in the nervous system of TRH are achieved in trace amount acting as a neuromodulator rather than endocrine hormones, even if the synthesis of TRH in the hypothalamus is disturbed or compensated, whether the total level of TRH in the brain is sufficient or excessive in maintaining its normal regulation functions is hard to assess. Therefore, the relationship between TRH and PD still needs further discussion. In conclusion, this study suggests TRH analog, Taltirelin possesses a protective effect on SH-SY5Y cells and rat primary midbrain neurons, as well as MPTP-induced subacute and rotenone-induced chronic PD mice models and this neuroprotective effect is associated with inhibition of apoptosis, ROS generation, phosphorylation of tau and α-synuclein, inhibition of AEP, thus providing evidence for applying a promising neuroprotective agent for treatment of PD.

## Author Contributions

CZ, GC, YX, ZZ, SP, KY and XC conceived and designed the study. CZ, GC, YT, WZ, QP, JW, CC, XY and SN performed the experiments. CZ and GC analyzed the data. CZ, GC, YX, SP, KY and XC wrote the manuscript. All authors read and approved the final manuscript.

## Conflict of Interest Statement

The authors declare that the research was conducted in the absence of any commercial or financial relationships that could be construed as a potential conflict of interest.
