# Supplementary material for: TRH Analog, Taltirelin Protects Dopaminergic Neurons From Neurotoxicity of MPTP and Rotenone
Source: Front Cell Neurosci. 2018 Dec 20;12:485. doi: 10.3389/fncel.2018.00485 (PMC6306470; doi:10.3389/fncel.2018.00485)
Supplement: Supplementary file 1 [file Data_Sheet_1.docx]

**Supplementary materials**

**TRH Analog, Taltirelin Protects Dopaminergic Neurons from Neurotoxicity of MPTP and Rotenone**

**Supplementary Figure**

**
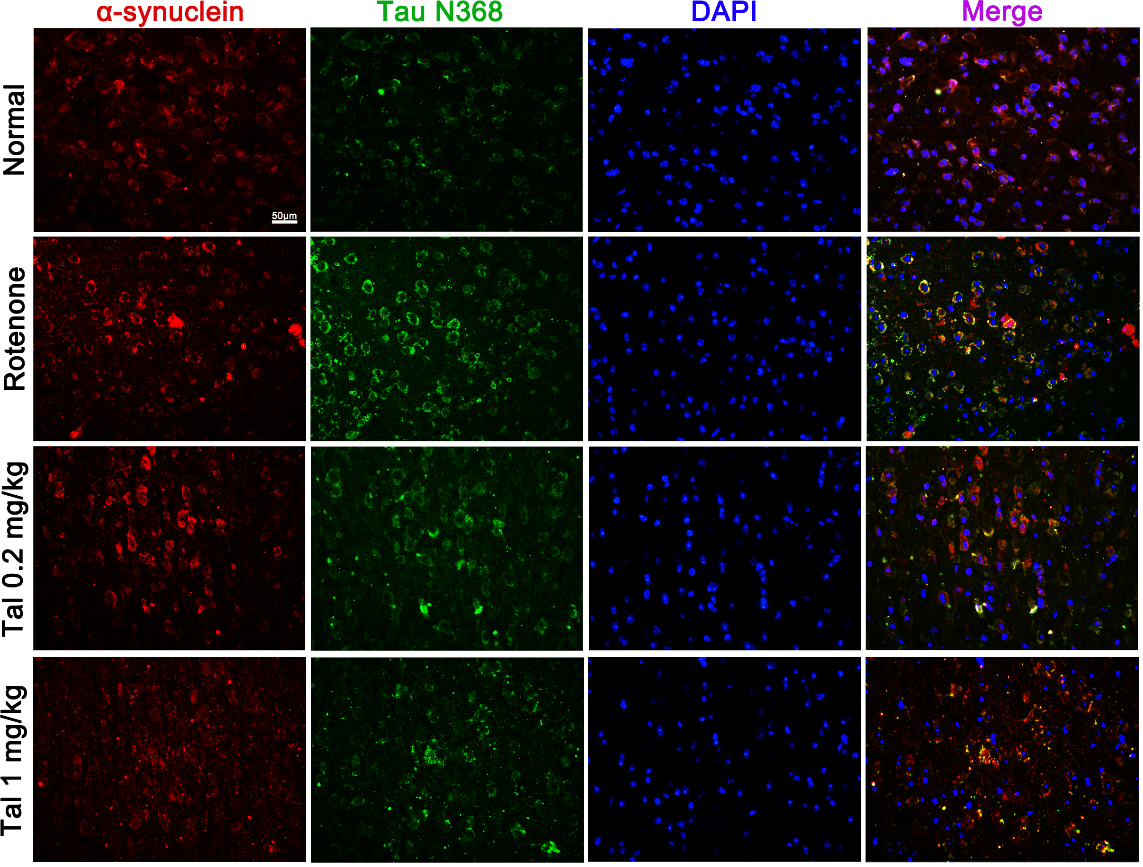
**

**Figure S1.** Double-label immunofluorescence images showed co-localization of α-synuclein and tau N368 in the striatum of each group (normal, rotenone, 0.2 or 1 mg/kg Taltirelin). Taltirelin, especially 1 mg/kg group had much fewer and more dispersive distribution of α-synuclein and tau N368 in the SN than that in rotenone group. *N* = 3.

**
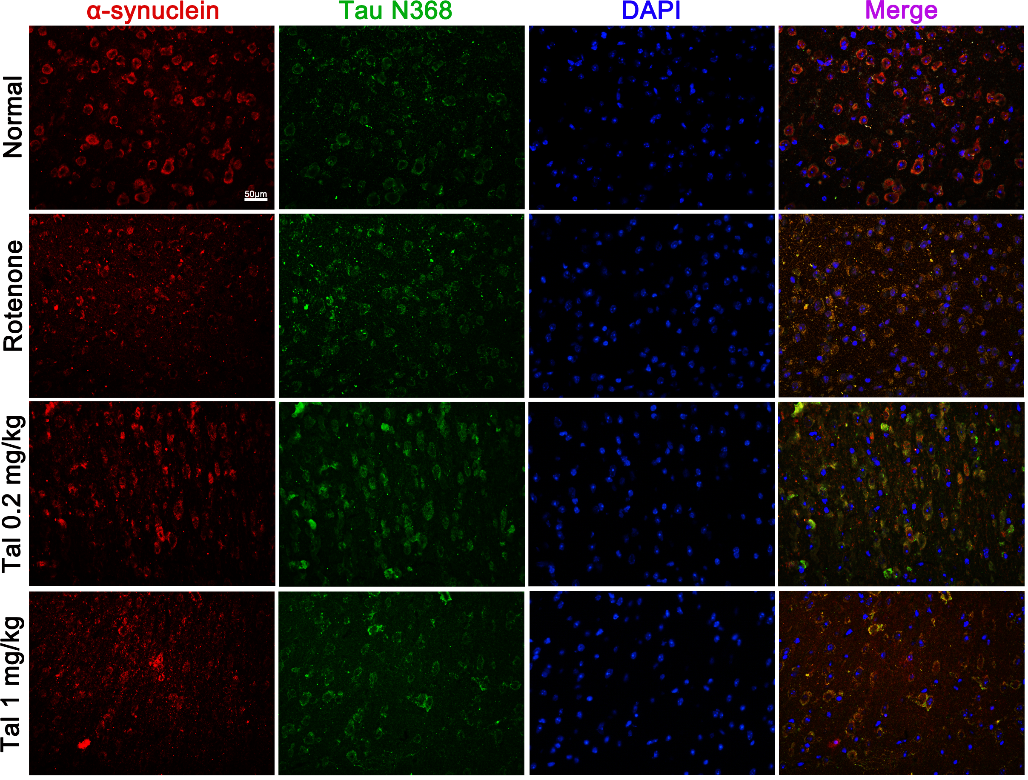
**

**Figure S2.** Double-label immunofluorescence images showed co-localization of α-synuclein and tau N368 in the cortex of each group (normal, rotenone, 0.2 or 1 mg/kg Taltirelin). No obvious difference of level or distribution were found among groups. *N* = 3.

**
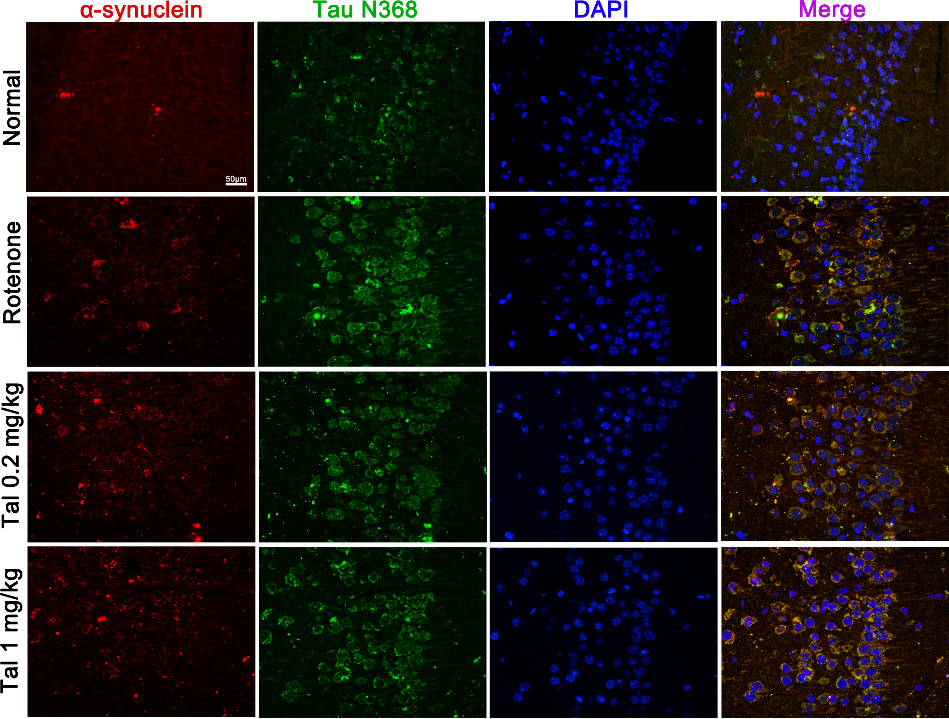
**

**Figure S3.** Double-label immunofluorescence images showed co-localization of α-synuclein and tau N368 in the hippocampus of each group (normal, rotenone, 0.2 or 1 mg/kg Taltirelin). No obvious difference of level or distribution were found among groups. *N* = 3.

**

**

**Figure S4. Measurement of Serum Thyroid Hormones in MPTP-induced PD Mice.** The subacute MPTP-induced PD model mice were given saline, 0.2, 1 or 5 mg/kg Taltirelin i.p. injection and blood was collected 2 h later. ELISA was used to measure the levels of different substances. **(A)** Serum thyroid stimulating hormone (TSH) concentrations; **(B)** Serum total triiodothyronine (TT3) concentrations; **(C)** Serum total thyroxine (TT4) concentrations; **(D)** Serum free T3 (FT3) concentrations; **(E)** Serum free T4 (FT4) concentrations. ***p* < 0.01 vs. control. *N* = 3. Error bars represent SEM.
